# Supplementary material for: Single-Cell RNA Sequencing Reveals Differences in Chromatin Remodeling and Energy Metabolism among In Vivo-Developed, In Vitro-Fertilized, and Parthenogenetically Activated Embryos from the Oocyte to 8-Cell Stages in Pigs
Source: Animals (Basel). 2024 Jan 31;14(3):465. doi: 10.3390/ani14030465 (PMC10854501; doi:10.3390/ani14030465)
Supplement: Supplementary file 1 [file animals-14-00465-s001.zip › animals-2807560-supplementary.pdf]

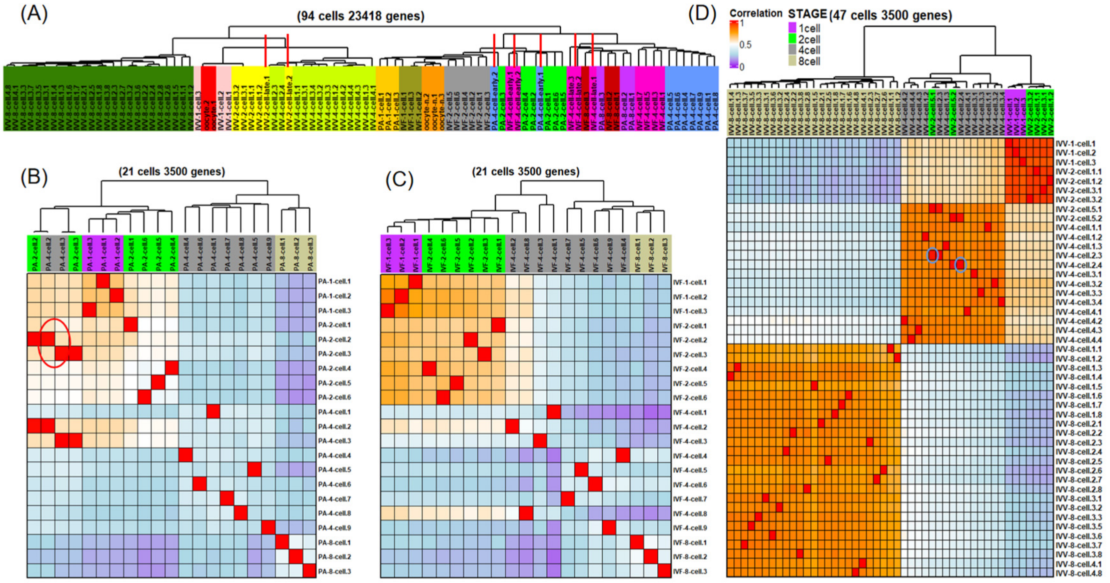

Supplementary Figure S1. Hierarchical Clustering and Correlation Analysis of Samples before Selection(A)Hierarchical clustering of single-cell RNA-seq from oocyte-8 cell stage of the three types of embryos (all 94 samples, with samples falling into adjacent stages marked with a red line). (B)Heatmap of expression levels of the top 3500 highly variable genes during the 1-8 cell stages within the PA group (column clustering). (C)Heatmap of expression levels of the top 3500 highly variable genes during the 1-8 cell stages within the IVF group (column clustering). (D)Heatmap of expression levels of the top 3500 highly variable genes during the 1-8 cell stages within the IVV group (column clustering).

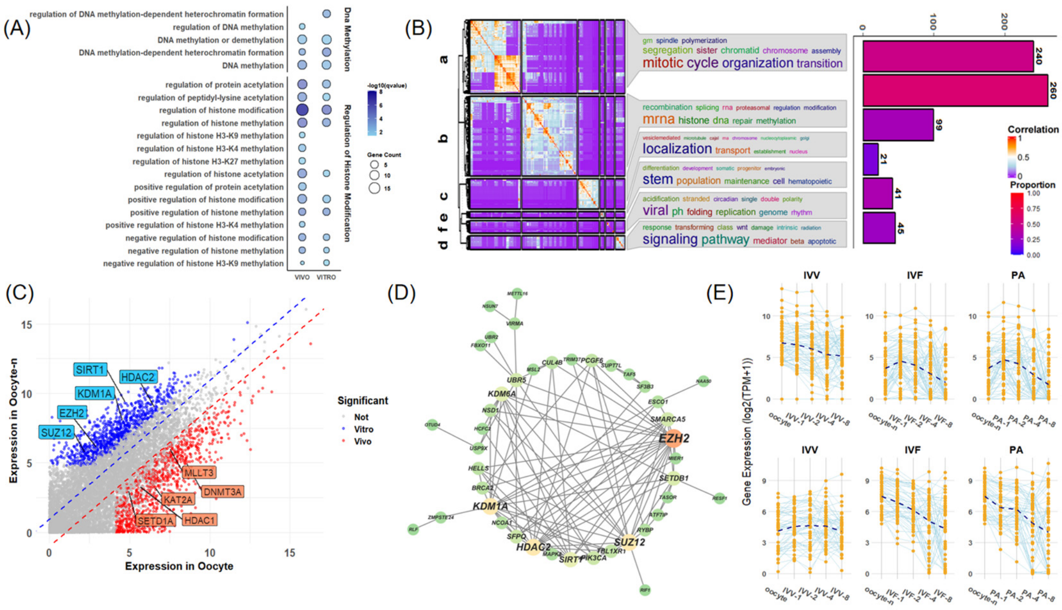

Supplementary Figure S2. Differential Gene Enrichment Analysis and Comprehensive Gene Screen.(A) Bubble chart showing GO terms

related to regulation of histone modifications and dna methylation from category C for *in vivo* matured oocytes and category B for *in vitro* matured oocytes, derived from GO enrichment and clustering of differentially expressed genes. (B)Left: GO terms from the *in vitro* maternal mRNA set, correlated with significantly expressed *in vitro* genes, are categorized into six distinct clusters (labeled a-f). Each cluster is visualized through a word cloud, emphasizing the prevalence of various biological processes. Right: A bar chart presents the number of GO terms and the proportion of associated genes within each category. (C) Scatter plot illustrating the correlation between gene expression levels(log2(TPM+1)) in *in vivo* and *in vitro* matured oocytes, with the top 5 genes from the PPI analysis of the 'remod' collections in both conditions labeled. (D) The PPI network plot for the 'remod' collection in *in vitro* matured oocytes. (E) From top to bottom: Expression trajectories of the 'remod' collections in *in vivo* and *in vitro* matured oocytes, respectively. The black line indicates the average expression level.

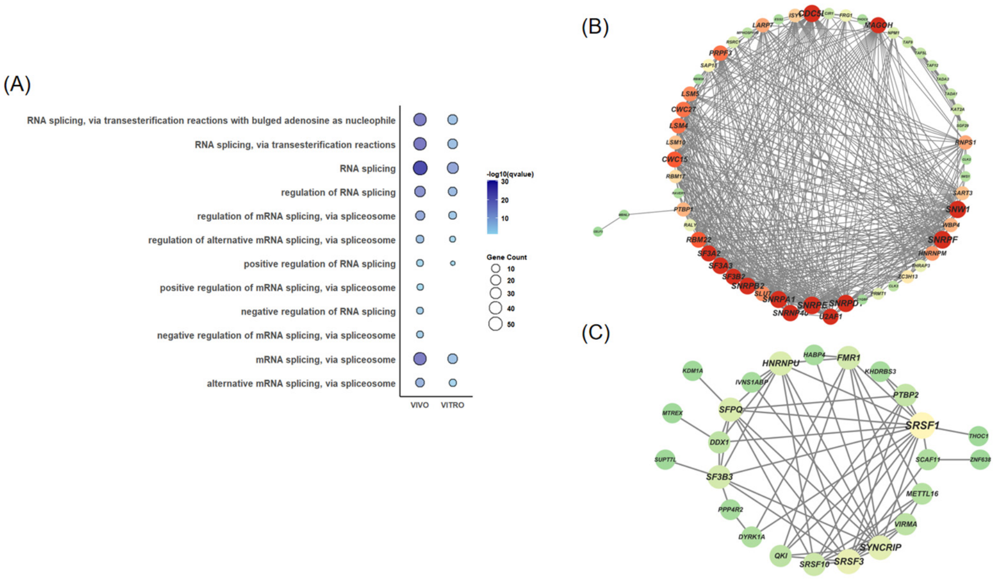

Supplementary Figure S3. Differential Gene Enrichment and PPI Analysis for mRna Splicing. (A) Bubble chart showing GO terms related to mRna splicing from category C for *in vivo* matured oocytes and category B for *in vitro* matured oocytes, derived from GO enrichment and clustering of differentially expressed genes. (B) The PPI network plot for genes from the *in vivo* section of the analysis, as highlighted in Panel A. (C) The PPI network plot for genes from the *in vitro* section of the analysis, corresponding to Panel A.

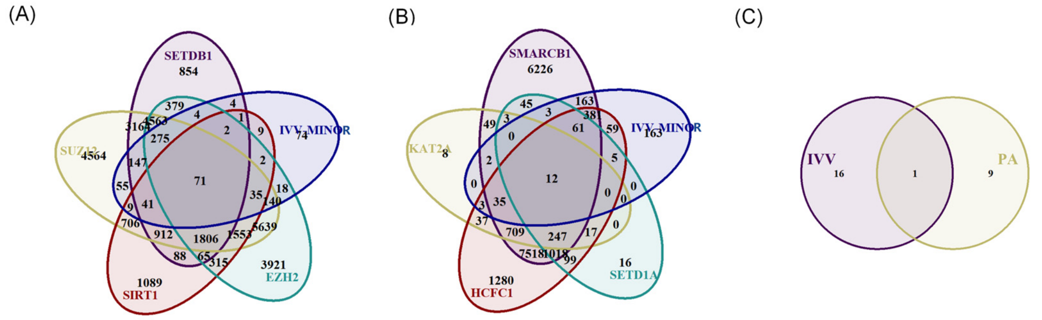

Supplementary Figure S4: Confirming Regulatory Connections between Core Genes and Minor ZGA. (A) Venn diagram illustrating the overlap of significantly upregulated maternal genes in the activation-related group (including *SMARCB1*, *HCFC1*, *KAT2A*, *SETD1A*) in in vitro matured oocytes, with genes independently upregulated during the first minor ZGA in IVV embryos. (B) Venn diagram showing the overlap of significantly upregulated genes in the silencing-associated group (including *EZH2*, *SUZ12*, *SETD1B*, *SIRT1*) in in vivo matured oocytes, with genes independently upregulated during the first minor ZGA in IVV embryos. (C) Venn diagram depicting the overlap of genes involved in the methylation of H3-K9, H3-K4, H3-K27, and the acetylation of H3-K14 in the in vivo maternal environment, with those involved in the minor ZGA of PA embryos.

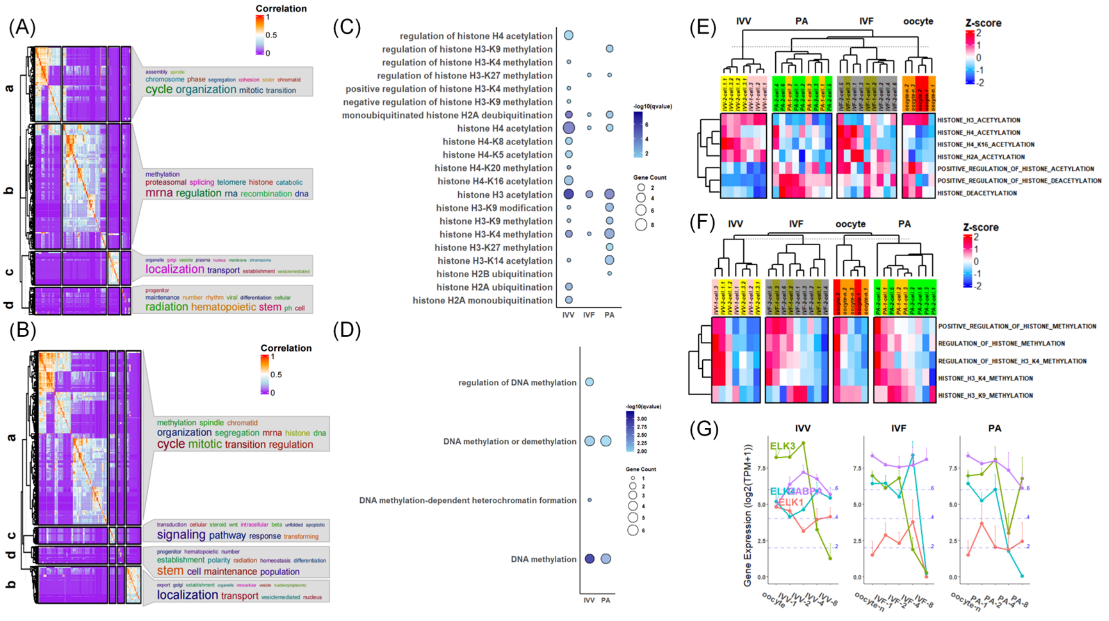

Supplementary Figure S5: Enrichment Analysis of Minor ZGA. (A) GO terms derived from the enrichment analysis of a combined gene set, including the minor ZGA gene set of IVF and the maternal mRNA set from in vitro matured oocytes. These terms were selected based on their correlation with genes significantly upregulated independent of PA and IVV, and were categorized into four distinct clusters (labeled a-d). Each cluster is represented by a word cloud, highlighting the prominence of different biological processes. (B) GO terms from the enrichment analysis of a combined gene set, encompassing the minor

ZGA gene set of PA and the maternal mRNA set from in vitro matured oocytes, selected based on their correlation with genes significantly upregulated independent of IVV and IVF. These terms were similarly categorized into four distinct clusters (labeled a-d), with each cluster represented by a word cloud emphasizing the key biological processes. (C) Bubble chart depicting GO terms related to histone modification among genes independently significantly upregulated during minor ZGA in IVV, IVF, and PA embryos. (D) Bubble chart illustrating GO terms associated with DNA methylation among genes independently significantly upregulated during minor ZGA in IVV, IVF, and PA embryos. (E) GSVA of GO terms related to histone acetylation from the oocyte to the 2-cell stage in IVV, IVF, and PA embryos. (F) GSVA of GO terms related to histone methylation from the oocyte to the 2-cell stage among IVV, IVF, and PA embryos. (G) From left to right: expression trajectories of *POU5F1*, *SOX2*, *KLF4*, *NANOG*, and *MYC* from the 1-8 cell stages in IVV, IVF, and PA embryos, respectively.

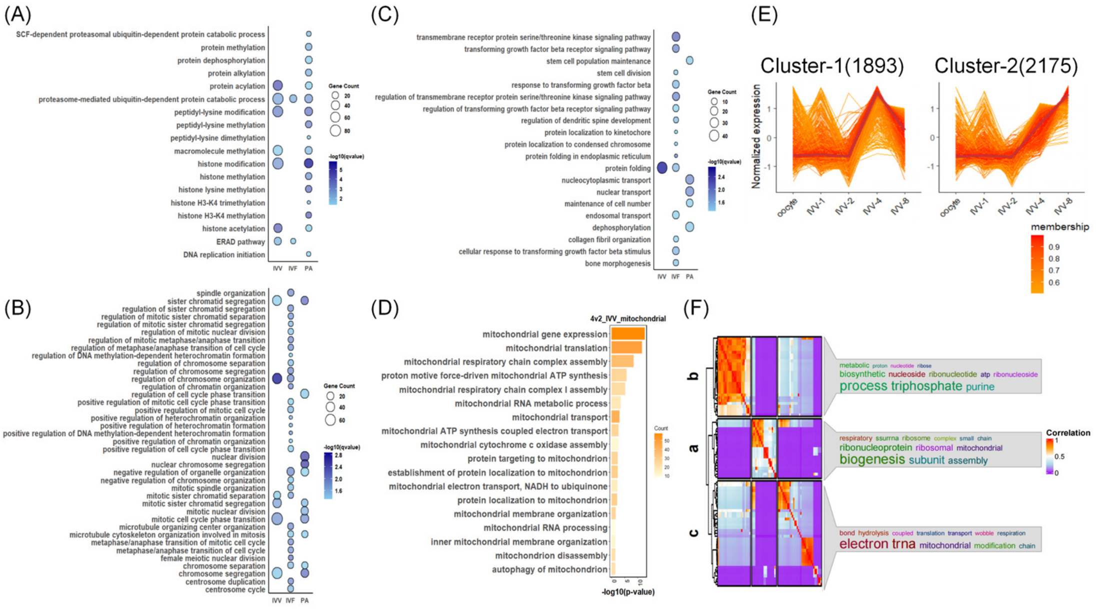

Supplementary Figure S6: Enrichment Analysis of Major ZGA. (A) Bubble chart showing GO terms mainly related to histone modifications among genes independently upregulated during the first major ZGA in IVV, IVF, and PA embryos. (B) Similar to Panel A, this chart focuses on GO terms related to the cell cycle. (C) Like Panel A, this chart displays GO terms associated with cell communication and substance transport. (D) The bar chart displays GO terms related to mitochondrial in Panel A of Figure.5(E) Clustering diagram categorizing expression patterns of significantly upregulated genes during the 2-4 cell stage in IVV embryos. (F) GO terms from the enrichment analysis of genes continuously upregulated from the 2-8 cell stage. These terms are categorized into 3 distinct clusters (labeled a-c), each depicted as a word cloud to highlight diverse biological processes.

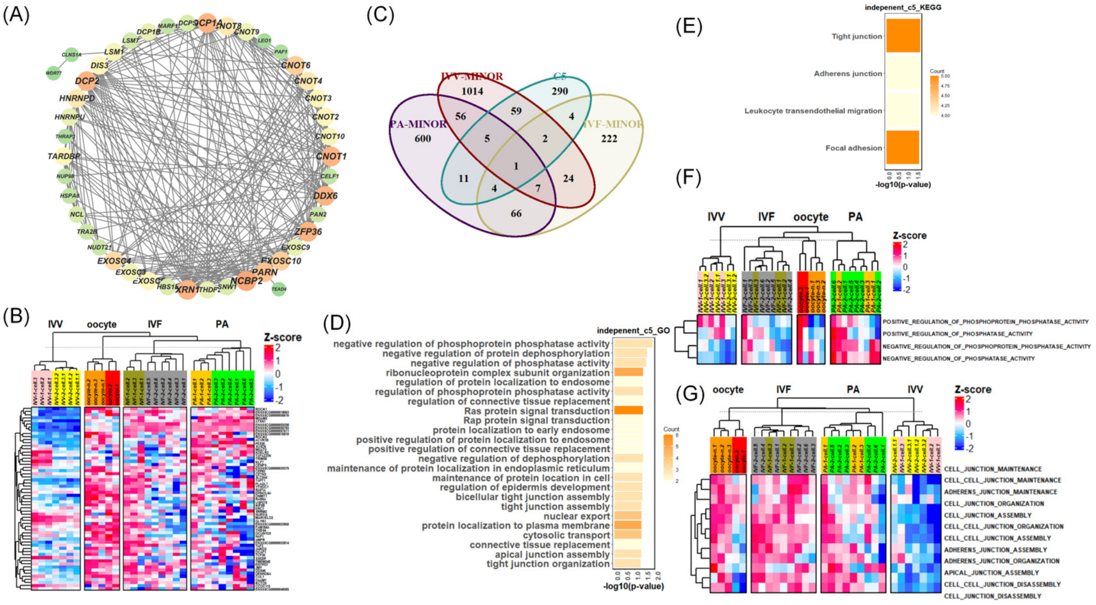

Supplementary Figure S7: Enrichment Analysis of Genes Related to mRna Decay of C5 During Minor ZGA. (A) PPI network displaying the interactions among genes in Cluster 2 from Panel C of Figure.6. (B) A heatmap of expression for genes in C5 from Panel C that independently significant downregulated in IVV embryos during minor ZGA, from oocyte to 2-cell stage(C) A Venn Diagram illustrates the overlap of downregulated gene sets (threshold: FDR < 0.05, log2FC < 0) in PA, IVF, and IVV embryos during the minor ZGA stage and the C5 gene set identified in Panel F of Figure.6. (D) The bar chart displays GO terms for genes in C5 from Panel C that independently significant downregulated in IVV embryos during minor ZGA. (E)The bar chart displays KEGG terms for genes in C5 from Panel C that independently significant downregulated in IVV embryos during minor ZGA. (F)GSVA of GO terms related to regulation of phosphatase activity from oocyte to 2-cell stage among IVV, IVF, and PA.(G)GSVA of GO terms related to DNA cell junction from oocyte to 2-cell stage among IVV, IVF, and PA.

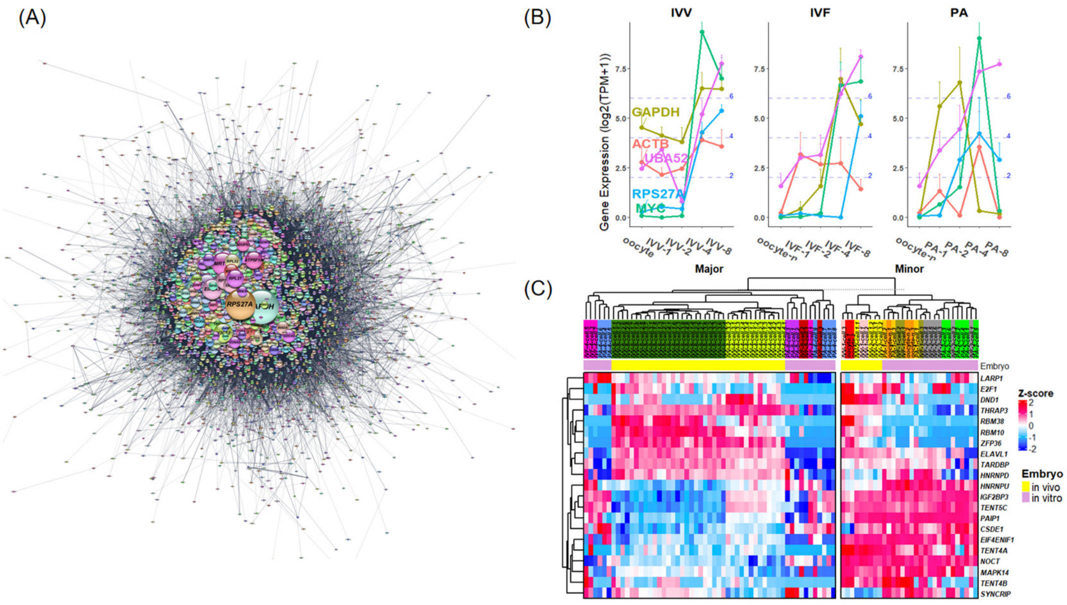

Supplementary Figure S8: Comparative Analysis of Decay of Key Genes during Major ZGA. (A) PPI network displaying the interactions among genes that significantly upregulated in IVV from 2-4 cell stage. (B) From left to right: expression trajectories of *GAPDH*, *ACTB*, *UBA52*,*MYC* and *RPS27A* from the oocyte-8 cell stages in the IVV, IVF, and PA embryos.(C) A heatmap of gene expression for maternal mRNA stabilizing-related genes from the oocyte to the 8-cell stage in three types of embryos: IVV, IVF, and PA.

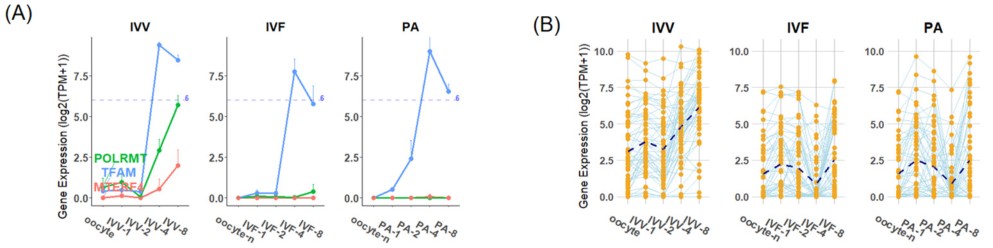

Supplementary Figure S9: Comparative Analysis of Up/Down stream Regulation of Mitochondrial Genes. (A) From left to right: expression trajectories of *POLRMT*, *TFAM* and *MTERF4* from the oocyte-8 cell stages in the IVV, IVF, and PA embryos.(B) From left to right: expression trajectories of nucleosomal components associated with the proteome from the oocyte-8 cell stages in the IVV, IVF, and PA embryos.The black line indicates the average expression level.

Stage	No. of embryos	No. of cells	Collection time (post-natural mating)
Oocyte	2	2	24 h (post-estrus)
1-cell	3	3	20 h
2-cell	4	7	36-40 h
4-cell	5	14	60-64 h
8-cell	5	26	84-90 h

Supplementary Table S1 The collection schedule and number of embryos for scRNA-seq
